# Establishment and ultrasound characteristics of atherosclerosis in rhesus monkey

**DOI:** 10.1186/1475-925X-14-S1-S13

**Published:** 2015-01-09

**Authors:** Wen Zeng, Xiaorong Wen, Li Gong, Jiayu Sun, Jing Yang, Jichun Liao, Can Qian, Wei Chen, Bin Song, Fabao Gao

**Affiliations:** 1 Sichuan PriMed Bio-tech Co., Ltd. Chengdu, China; 2 Department of Ultrasonography, West China Hospital, Sichuan University, Chengdu, China; 3 Department of Radiology, West China Hospital, Sichuan University, Chengdu, China; 4 Department of Anesthesiology, West China Hospital, Sichuan University, Chengdu, China

## Abstract

**Background:**

Atherosclerosis is one of the main risk factors cause acute cerebral-cardio vascular diseases. It's of great significance to establish an atherosclerosis animal model that can mimic the characteristics and nature course of human patients. Therefore, a rhesus monkey model was induced by high-fat diet to monitor their lipid profile and intima-media thickness (IMT) of artery walls and study atherosclerosis progression.

**Methods:**

Fifty male rhesus monkeys were enrolled in this study. All of these monkeys were aged 7 to 14 years with BMI >30 kg/m^2^. They were fed with high-fat diet containing 10% of fat for the first 48 weeks. Use ultrasound to measure the IMT at bilateral common carotid arteries and their bifurcations and aorta (AO) of the monkeys, and screen out the individuals with thickened IMT for the next phase. In the next 48 weeks, some of these monkeys (n = 4) were fed with standard diet containing 3% fat. Meanwhile the other monkeys (n = 5) were fed with high-fat diet for another 48 weeks. Their serum lipid level was monitored and arterial IMT was also determined periodically.

**Results:**

Serum lipid level of all 50 monkeys elevated after fed with high-fat diet for the first 48 weeks. IMT thickening at right common carotid bifurcation and aorta (AO) was thickened in 9 monkeys. Furthermore, 4 of these 9 monkeys were fed with standard diet and other 5 monkeys were fed with high-fat diet in the following 48 weeks. The serum lipid level of the 4 monkeys recovered and their IMT at RBIF and AO did not progress. However, the lipid level of other 5 monkeys remained high, and their IMT thickening of AO progressed, and plaques and calcification focuses were found at the anterior wall of aorta near the bifurcation of common iliac artery.

**Conclusions:**

After high-fat diet induction for 96 weeks, serum lipid levels of rhesus monkeys elevated significantly, which subsequently caused IMT thickening and plaques formation. When IMT thickening occurred, further vascular injury may be prevented by reducing diet fat content. Our study indicates that vascular injury of high-fat diet induced rhesus monkey is similar to that of human in position and progression.

## Introduction

Cerebral-cardio vascular disease is the biggest threat to human health [1,2] and atherosclerosis is one of the main reasons that cause this critical disease. As atherosclerosis progressing, main arteries are vulnerable to form plaques. Defluxion of unstable plaques would lead to vascular thrombosis of downstream target organs, thereby causing acute cardiovascular and cerebrovascular events. Due to plaque disruption has great uncertainty, it is very important to establish an animal model that can adequately simulate the plaque formation like human patients, and can be used for development and research of diagnostic and therapeutic drugs.

Most of the reported animal models of atherosclerosis are rodents [3]. But atherosclerotic plaque is a chronic and complex disease caused by multiple factors and there are significant differences between rodents and human in lipid metabolism, plaque components, plaque formation and position. Compared with other animal models, rhesus monkeys are more similar to human in physiology and the susceptibility of metabolic disease [4]. Because of high similarities with human in genes, histological structures, immunology, physiology and metabolism with human, rhesus monkey became the most widely used non-human primate animal in medical research [5].

Since 1950, there have been few of laboratories successfully inducing atherosclerosis in monkey. Multiple studies have indicated the influences of different contents of diet on the development of atherosclerosis in rhesus monkey, and analyzed the composition of lumens and plaques by using autopsy [6-9]. However, besides the use of high-cholesterol diet would largely affect the animal's health, the lipid level of animals induced by high-cholesterol diet is not stable. Additionally, high-sugar diet is not like the normal food of patients, and using autopsy to identify the presence of atherosclerosis and plaques affects the preservation and application of models. All of these are problems existing in previous researches.

Ultrasound, which can clearly picture the main arteries, particularly conditions of carotid artery walls, has been used as a non-invasive method in recent years to detect atherosclerosis at an early stage [10]. It can not only increase the predictability of cardiovascular diseases [11], but also act as an alternative maker to determine the extent of atherosclerotic progression [12]. Atherosclerotic imaging, the IMT measurement as a benchmark, has become the basic reference of the research and development of cardiovascular drugs [13].

In this study, we used non-cholesterol high-fat diet, selected non-invasive B-mode ultrasound to monitor and identify the model periodically, and compared the progression of IMT and change of serum lipid level. Hoping that our rhesus monkey model with plaques can be applied for atherosclerosis and plaque related researches.

## Methods

### Agents and apparatus

Ketamine hydrochloride (10 mg/ml, Bioniche Teoranta); Electronic weighing scale (Yousheng XK3123); Electronic balance (Yousheng BS2100+); Color Doppler ultrasound apparatus (SonoSite M-turdo); Automated biochemistry analyzer Synchron CX4 PRO (Beckman Coulter, Brea, Calif) and all blood chemistry detection reagents were bought from Beckman Coulter Inc.

### Animals

All monkeys enrolled in this study were provided by Ya'an PriMed Bio-tech Co., Ltd. After examination, under the condition that other diseases were excluded, 50 middle-aged male rhesus monkeys (aged 7-14 years, and BMI > 30 kg/m^2^) were enrolled in this study. At baseline, all monkeys had total cholesterol (TC) concentrations < 4 mmol/L, triglyceride (TG) concentrations < 1 mmol/L, and low density lipoprotein (LDL-c) concentrations < 2.5 mmol/L.

### Diets

**Figure 1:**
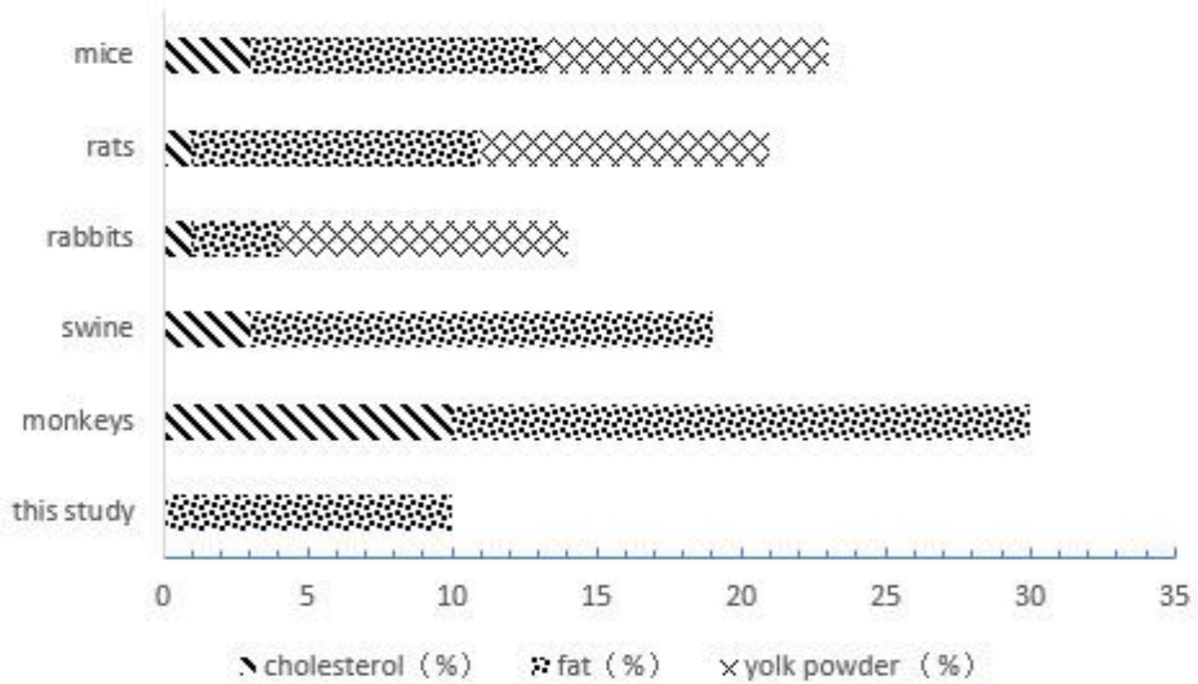
**Profile of fat content in high-fat diets for establishing atherosclerosis animal models with different species**.

There are 2 kinds of diets involved in this study: high-fat diet (containing 18% protein, 58% carbohydrate, 14% water and 10% fat) and standard diet (containing 18% protein, 69% carbohydrate, 10% water and 3% fat), fat percentage of the high-fat diet used in this study is shown in Figure 1 (compared with other species in atherosclerosis modeling). Approximately 250 g feed was supplied to each animal (available *ad libitum*) in the mornings, and apples or vegetable with equal nutrients were provided in the afternoons. Tap water was used as drinking water, which was taken freely by monkeys via the automatic bubbler.

### Quarantine

Animal quarantine procedures included physical examinations, intradermal skin test for mycobacterium tuberculosis, fecal examination for parasites, bacterial culture for salmonella, shigella, and serology for herpes B virus. The results of all 50 monkeys were negative.

### Maintenance

Monkeys were raised in stainless steel pair-housing monkey cages (2 m long, 1.7 m wide, and 2 m high with swing and woods), in a feeding room with the temperature of 19°C-26°C (66°F-79°F) and humidity of 50% ± 20%. The rate of ventilation was 10 times/h and the lighting cycle was 12 (day)/12 (night) hours. The monkeys were maintained in conformity with the requirements of "the National Institutes of Health Guide for the Care and Use of Laboratory Animal" of the United States, and all experimental protocols were reviewed and approved by the Animal Welfare and Use Committee of Sichuan PriMed Bio-tech Co., Ltd.

All the monkeys had been domesticated for at least 2 years. Before this study, there was 4-week long acclimation period in order that all monkeys could cooperate with the experimenters during the trial.

### Trial Arrangements

**Figure 2:**
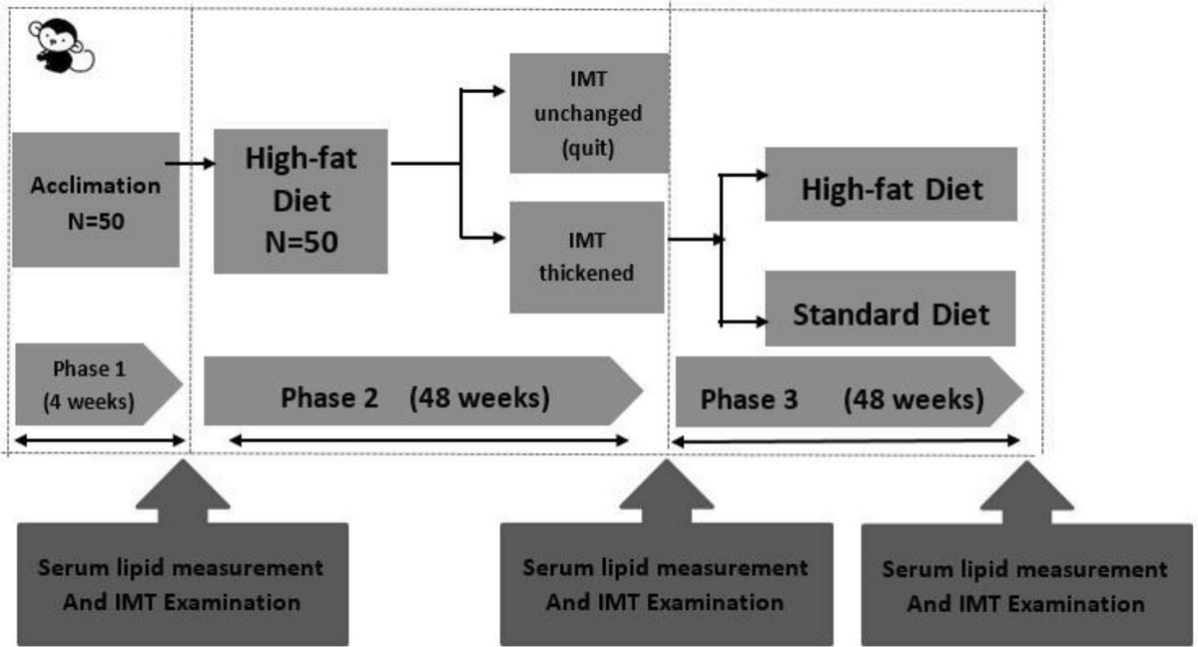
**Trial flow chart**.

As Figure 2 shows, this study lasted for approximately 2 years and was divided into 3 phases. Phase 1 is a 4-week long acclimation period. At the end of the acclimation period, the lipid parameters of all animals were measured. Meanwhile, the intima-media thickness of abdominal aorta and bilateral common carotid arteries and their bifurcations were measured. Phase 2 is a high-fat diet induction period which lasted for 48 weeks. In phase 2, all 50 animals were fed with high-fat diet, and at the end of this period the lipid profile and IMT were measured in order to acquaint the influence of this composition of high-fat diet on serum lipid and screen out the individuals with thickened IMT. Only individuals with thickened IMT were enrolled in phase 3. Phase 3 lasted for another 48 weeks, in this period, monkeys with thickened IMT were divided into 2 groups randomly. Monkeys in one group were fed with high-fat diet while the other group were fed with standard diet instead. Similarly, lipid profile and artery IMT were measured at the end of phase 3.

### Lipid levels measurement

Blood samples were collected at cephalic veins after fasting for 16 hours after fasting. TC, TG and LDL-c levels were measured by enzymatic methods using an automated biochemistry analyzer.

### Ultrasound examination

**Figure 3:**
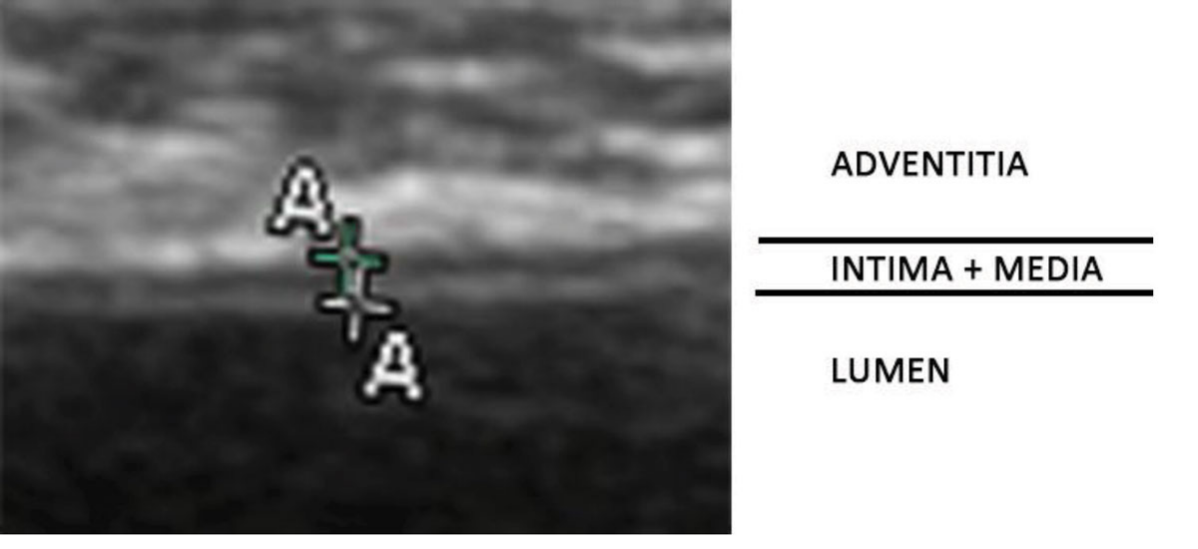
**A typical double line pattern ultrasound image of a normal abdominal aortic artery wall of a monkey**. The distance between the centers of the two crosses represents the distance between the inner and the outer echogenic lines and corresponds to the ultrasound image of intima-media thickness.

After fasting for 8 hours, monkeys were sedated by an intramuscular injection of 10% ketamine hydrochloride and ultrasound examinations were performed with the use of an 8-MHz ultrasonic probe by a single trained sonographer who was kept unaware of the trial information. Monkeys were examined in the supine position with their heads slightly extended. With this technique, 2 parallel echogenic lines separated by an anechoic space can be visualized at levels of the artery wall. It was previously proved that these lines were generated by the blood-intima and media adventitia interfaces [14]. We define the IMT value as the distance from the adventitia-media boundary of the near wall to the lumen-intima and media adventitia interfaces of the far wall following the typical double line pattern (Figure 3) [15]. IMT values of 3 different projections: anterior, lateral, and the bifurcation, were averaged to obtain the mean IMT. An atherosclerotic plaque was defined as a distinct area where the intima-media encroached into the vessel lumen and where its thickness was at least 50% greater than that of the adjacent sites. When an atherosclerotic plaque was present, it was included in the measurements of IMT [16].

#### Carotid artery

When determining one side of carotid arteries, adjust the head of the monkey 45° to the opposite side. Longitudinally view the common carotid artery and bifurcation with a superficial probe, freeze and amplify the image when it shows clearly the intima of anterior and posterior wall of artery so that IMT values could be determined.

#### Abdominal aorta

About 1.0 cm left to the medioventral line, longitudinally view the whole aorta (from diaphragm to the bifurcation of iliac artery). Observe from multi-section and multi-angle if the monkey is obese or filled with gas in the abdominal cavity.

### Statistical analysis

All data were expressed as mean±SD. Differences among groups were compared by analysis of variance. Difference before and after treatment were compared by paired t-test. All statistical analysis were performed using SPSS statistics 19.0 software and a P value less than 0.05 was considered statistically significant.

## Results

### Phase 1

**Figure 4:**
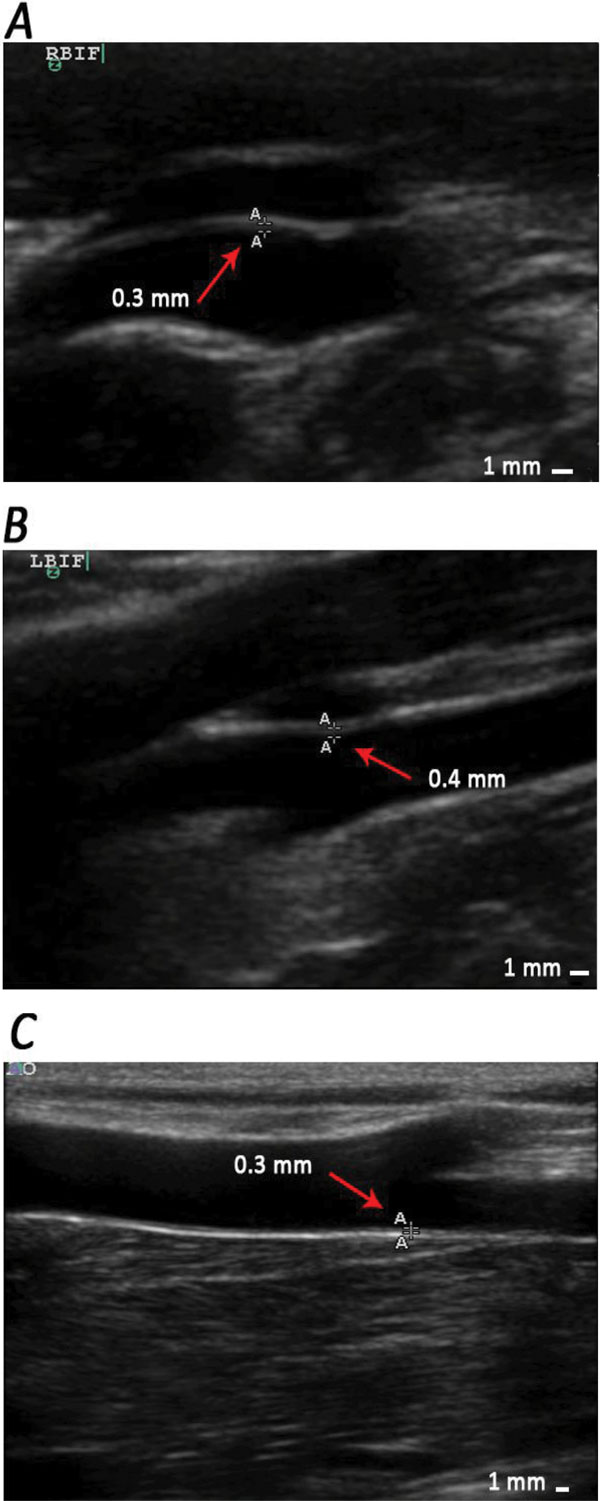
**Typical ultrasound images of normal carotid arteries and aortas of different rhesus monkeys**. A, B and C show images of normal intima plus medial thickness measurement of RBIF, LBIF and AO, respectively. The values of IMT in picture A, B and C are 0.3 mm, 0.4 mm and 0.3 mm respectively.

To acquire the baseline, we assayed the serum lipid related parameters of all 50 monkeys. IMT of different positions were measured by ultrasound. The mean values of lipid parameters are as below: TC = 3.12 ± 0.64 mmol/L, TG = 0.57 ± 0.17 mmol/L, LDL-C = 1.31 ± 0.24 mmol/L (n = 50). The mean values of IMT determined at different artery positions are as below: IMT (RCCA) = 0.36 ± 0.08, IMT (RBIF) = 0.40 ± 0.05, IMT (LCCA) = 0.34 ± 0.06, IMT (LBIF) = 0.40 ± 0.01, IMT (AO) = 0.42 ± 0.09 (Figure 4 shows normal IMT value measured at different positions).

### Phase 2

After induced by high-fat diet for 48 weeks, levels of lipid related parameters of these 50 monkeys all elevated significantly: TC = 5.2 ± 0.79 mmol/L, TG = 1.1 ± 0.31 mmol/L, LDL-C = 2.5 ± 0.81 mmol/L (n = 50, p < 0.05). Among these monkeys, 9 monkeys showed thickened IMT at common carotid artery bifurcation and abdominal aorta: IMT-RBIF =0.58 ± 0.21, IMT-AO = 0.68 ± 0.29 (n = 9, p < 0.05). The IMT profile and serum lipid profile of these 9 monkeys are shown in table 1 and table 2. Other 41 monkeys were dropped out from the trial.

**Table 1:** IMT profile of 2 groups of rhesus monkeys at the end of each phase.

IMT (mm)	high-fat diet for 1 year & standard diet for 1 year (n = 4)			high-fat diet for 2 years (n = 5)		
		
	phase 1	phase 2	phase 3	phase 1	phase 2	phase 3
RCCA	0.33 ± 0.10	0.35 ± 0.13	0.38 ± 0.10	0.39 ± 0.05	0.40 ± 0.07	0.42 ± 0.04
RBIF	0.38 ± 0.05	0.58 ± 0.10*	0.58 ± 0.10*	0.41 ± 0.05	0.51 ± 0.06*	0.60 ± 0.12#
LCCA	0.33 ± 0.06	0.33 ± 0.05	0.35 ± 0.06	0.34 ± 0.06	0.38 ± 0.04	0.38 ± 0.04
LBIF	0.40 ± 0.00	0.45 ± 0.06	0.48 ± 0.05	0.40 ± 0.02	0.46 ± 0.05	0.46 ± 0.05
AO	0.34 ± 0.06	0.55 ± 0.06*	0.55 ± 0.06*	0.48 ± 0.04	0.68 ± 0.11*	1.12 ± 0.23#

*p < 0.05, compared with phase 1

#p < 0.05, compared with phase 2

**Table 2:** Serum lipid profile at the end of each phase.

serum lipid parameters (mmol/L)	high-fat diet for 1 year & standard diet for 1 year (n = 4)			high-fat diet for 2 years (n = 5)		
		
	phase 1	phase 2	phase 3	phase 1	phase 2	phase 3
TC	3.42 ± 0.74	5.35 ± 0.87*	3.12 ± 0.64#	3.08 ± 0.84	5.04 ± 0.60*	4.91 ± 0.14*
TG	0.66 ± 0.25	1.15 ± 0.35*	0.71 ± 0.15#	0.55 ± 0.12	1.06 ± 0.28*	1.37 ± 0.25*
LDL-C	1.41 ± 0.22	2.50 ± 0.88*	1.58 ± 0.64#	1.28 ± 0.31	2.52 ± 0.76*	2.17 ± 0.26*

*p < 0.05, compared with phase 1

#p < 0.05, compared with phase 2

### Phase 3

**Figure 5:**
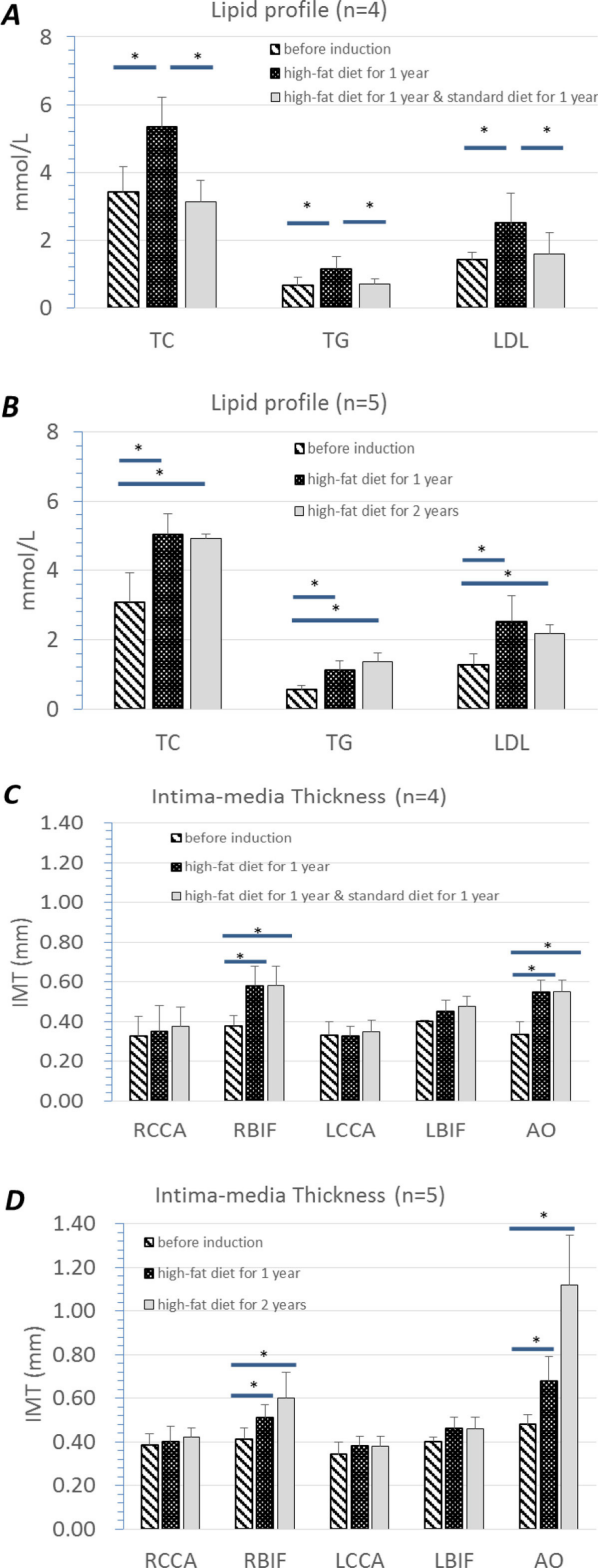
**Lipid and IMT profiles of the 9 monkeys with thickened IMT throughout the trial**. Picture A and B show the lipid profile. Picture C and D show the IMT profile. * p < 0.05.

**Figure 6:**
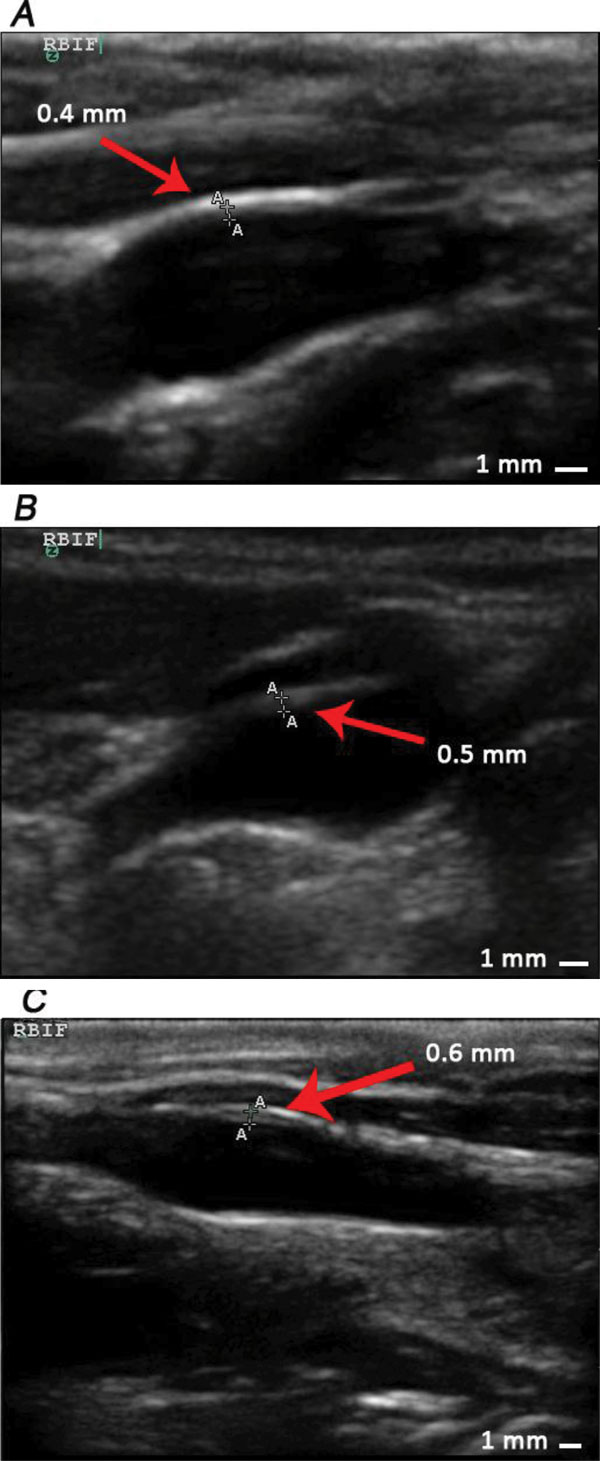
**Progress of IMT thickening at AO of one monkey with the No. 4353#**. A, B and C represent results measured at the end of phase 1,2,3 respectively, and the values of IMT in picture A, B and C are 0.5 mm, 0.6 mm and 0.8 mm respectively.

**Figure 7:**
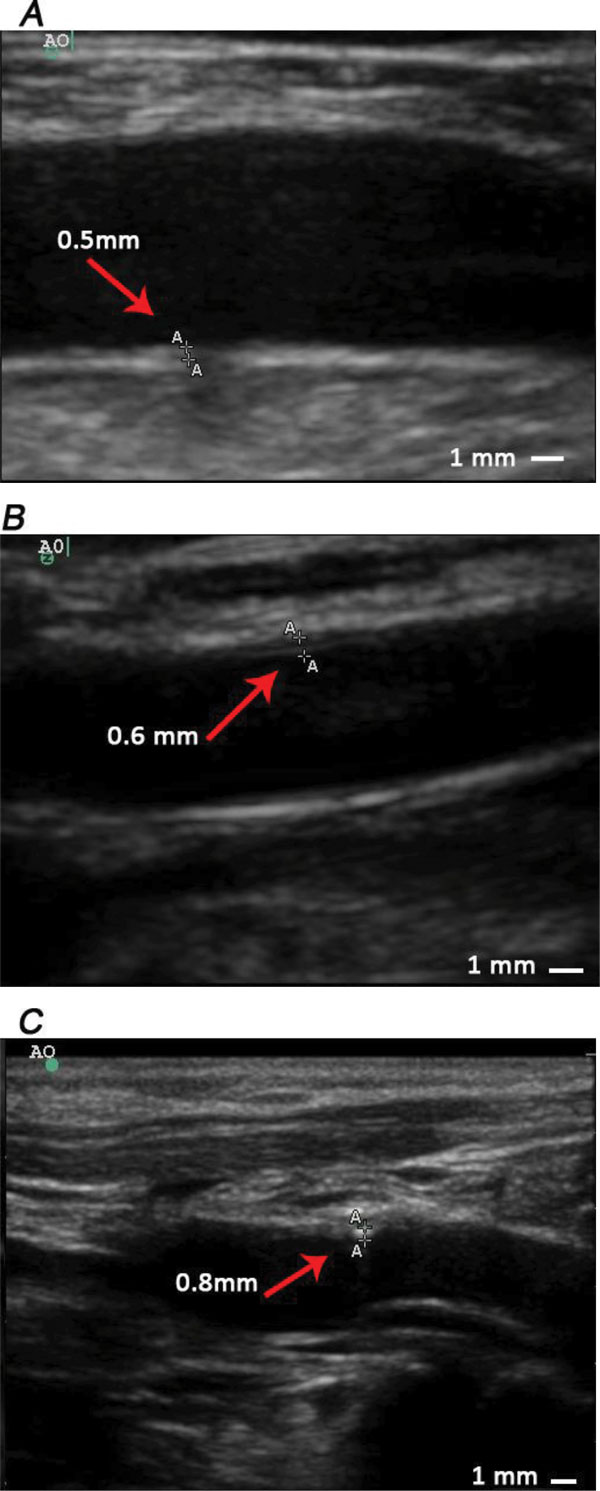
**Progress of IMT thickening at RBIF of one monkey with the No. 4353#**. Picture A, B and C represent results measured at the end of phase 1, 2, 3 respectively, and the values of IMT of picture A, B and C are 0.4 mm, 0.5 mm and 0.6 mm respectively.

**Figure 8:**
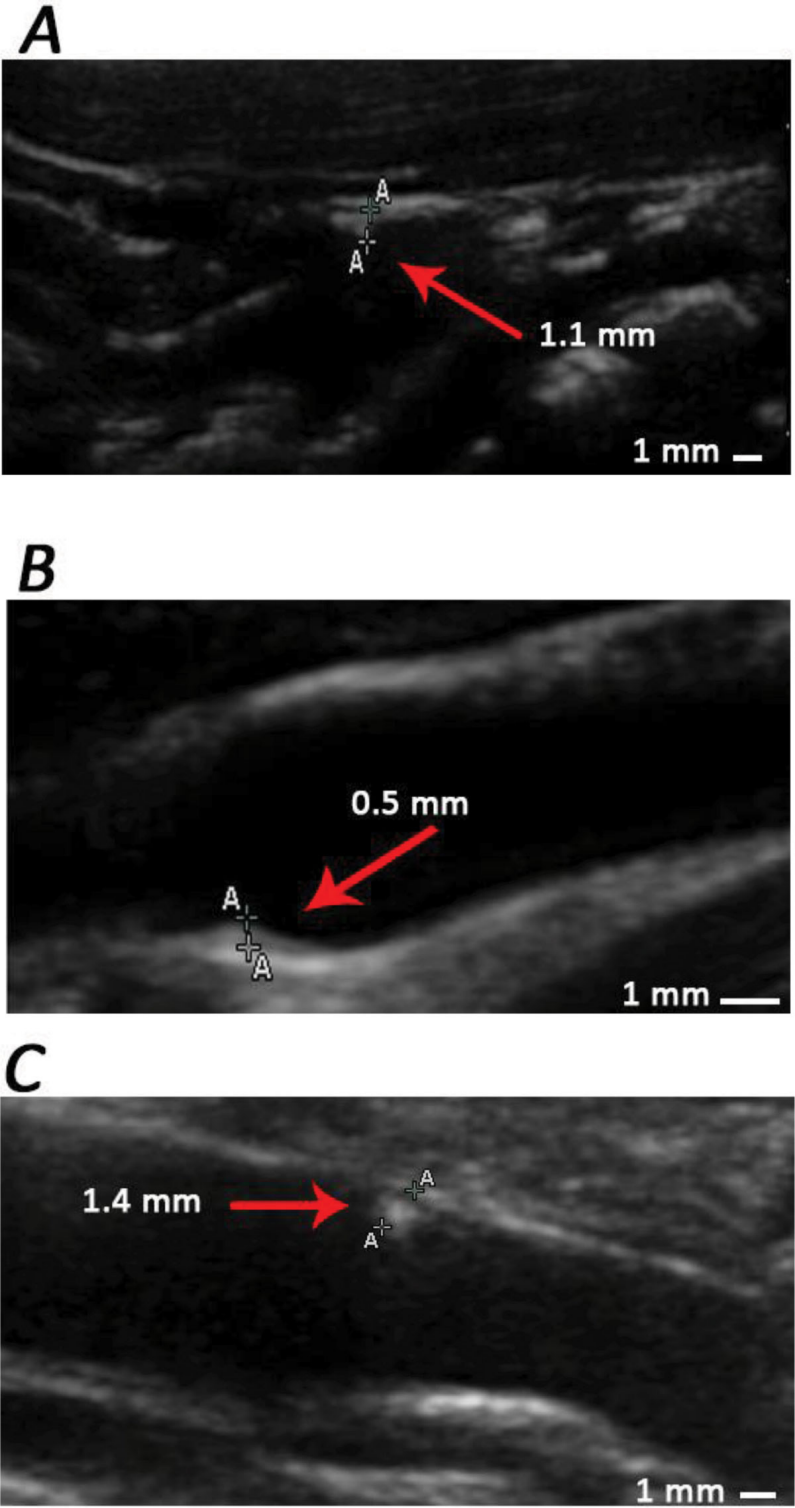
**Ultrasound images of calcification focuses found at different positions of monkey arteries**. A: a calcification focus at AO, near the bifurcation of common iliac artery. B: a calcification focus at LBIF. C: a calcification focus at RBIF.

At the beginning of phase 3, we divided those 9 monkeys into 2 groups: 4 monkeys in group 1 and 5 monkeys in group 2. Group 1 were induced by standard diet in the following 46 weeks, while group 2 were sequentially fed with high-fat diets. Similarly, lipid related parameters and IMT of those 9 monkeys were determined at the end of phase 3 (See Figure 5, table 1 and table 2). All serum lipid parameters of animals in group 1 decreased to normal range, while the parameters of animals in group 2 remained at a high level. Moreover, IMT values of animals in group 1 measured at all positions did not show further changes. Nonetheless, in animals of group 2, abdominal aorta IMT thickening and RBIF IMT thickening progressed (Figure 6, Figure 7) and plaques as well as calcification focuses were found at the anterior wall of aorta near the bifurcation of common iliac artery (Figure 8).

Shown from the results of this trial, long-term high-fat diet causes elevation of serum lipid levels of rhesus monkeys, furthermore, impels IMT thickening and plaque formation. The most severe position affected by this damage is the aorta, especially position near the bifurcation of common iliac artery, then the carotid bifurcations (Table 3). After adjusting the ingredients of the diets, standard diet with relatively low fat content could ameliorate high blood lipid level, while intake of high-fat diet sequentially could not only keep the serum lipid at a high level but also impel IMT thickening progressing.

**Table 3:** Record of plaques at different positions at the end of this trial.

position	RCCA	RBIF	LCCA	LBIF	AO
plaque	--	2	--	1	5

## Discussion

To establish atherosclerosis animal models, researchers have studied the effects of different lipid content diets on different species. Diets were added with certain amount of cholesterol in most of the modeling studies (Figure 1). High cholesterol diets elevate animal serum lipid levels rapidly. Atherosclerosis model established by high-cholesterol diets cannot completely mimic the nature course of human atherosclerosis. Moreover, the use of high-cholesterol diet would largely affect the animal's health, and the lipid level of animals induced by high-cholesterol diet is not stable. The high-fat diet used in this experiment was free from cholesterol and contained only 10% fat. This ingredient is relatively mild in establishing atherosclerosis in rhesus monkeys, while a diet consisting of 26% lipids, 22% proteins, and 52% carbohydrates were considered as low-fat diet in human [12].

Formation of human atherosclerotic plaque is a chronic long-term process in contrast with establishing animal models such as rodents, swine, etc., which costs only a few months. We have noticed that the IMT progression rate of our rhesus monkeys was slower than other animal species in atherogenic modeling. While in human atherosclerosis study, in patients with type 2 diabetes, an average annual increase of IMT 0.02 mm/year has been reported [17,18]. However, Jagdip et al. reported the common carotid artery IMT progression in coronary artery disease human patients were 0.0031 mm/48 weeks [19]. The average annual progression rates of IMT at different locations of monkeys in this study is similar to human patients (table 4). It is a chronic modeling process that we induced rhesus monkeys with mild high-fat diet for 2 years. During the induction period, we can monitor vascular changes dynamically. And the rhesus monkey model we established is more similar to patients, which might be more suitable for biomedical research.

**Table 4:** Average annual progression rates of IMT at different positions of monkey arteries.

location	IMT progression rate (mm/year)
RCCA	0.015
RBIF	0.095
LCCA	0.02
LBIF	0.03
AO	0.32

Early stage atherosclerosis is only characterized by the thickening of artery wall. Researchers compared the ultrasound and pathological methods, and found that ultrasound could accurately measure the arterial IMT, and the results obtained via ultrasound correspond well with pathological determination. The changes of IMT during atherosclerosis appeared earlier than the formation of plaques [11,20]. Among 50 rhesus monkeys enrolled in this study, 9 individuals showed IMT thickening, 5 formed plaques in different positions of arteries. The IMT of all animals formed plaques were thickened at certain degree, which accords with clinical features of human patients. Meanwhile, the sites of plaques mostly occurred at the bifurcation of bilateral common carotid arteries and the bifurcation of aorta close to common iliac artery, which highly resemble the clinical situation of patients (See Figure 8).

There were many studies focused on identifying risk factors contributing to atherosclerosis and coronary heart diseases (CHD). The Framingham Study developed a risk score that includes major risk factors, such as age, blood pressure, cigarette smoking, total cholesterol and diabetes status [21]. A similar score was developed that demonstrates the ability of traditional risk factors to predict CHD in the Atherosclerosis Risk in Communities (ARIC) study [22]. Other than TC and age, monkeys in this trial were free from other atherogenic risk factors. Monkeys in this study were middle-aged, which may be one of the reasons why the monkeys with preserved IMT were not so susceptible to high-fat diet. However, genetic polymorphism contributing to atherosclerosis and cardiovascular incidence is another type of emerging risk factor [23]. Similarly, family studies have demonstrated both mean and maximum measurements of CCA IMT are heritable [24]. The Northern Manhattan Family Study also indicated that the heritability for total carotid IMT, CCA and carotid bifurcation IMT were significant [25]. All 50 monkeys in this study showed elevated lipid level, but only 9 monkeys showed increased IMT after 48 weeks high-fat diet induction. This phenomenon indicates there may be heritability for CCA IMT, carotid bifurcation IMT and aorta IMT in rhesus monkeys.

## Conclusion

In rhesus monkey, levels of serum lipid-related parameters such as TC, TG and LDL-c may significantly elevate after being induced by high-fat diet which contents 10% of fat for 48 weeks. Part of these monkeys with elevated lipid levels show significantly thickened IMT at right common carotid bifurcation and aorta, which can be determined by ultrasound accurately. Continue to feed standard diet which contents 3% of fat to part of these monkeys with thickened IMT for another 48 weeks may neither ameliorate IMT thickening nor deteriorate, while serum lipid profile was benefited. However, for the rest of them fed with high-fat diet for the following 48 weeks sequentially, the IMT thickening at abdominal aorta progressed, meanwhile, their serum lipid remained at a high level. Since IMT at different positions could be determined by ultrasound which is a non-invasive method, and IMT act as an alternative maker to determine the extent of atherosclerotic progression, we can not only validate our atherosclerosis rhesus monkey model easily, but also apply it on new drug evaluation.

## Competing interests

Other than the grants listed in the acknowledgement section, the authors declare that they have no other competing interest.

## Ethics statement

The protocol was approved by the Institutional Animal Care and Use Committee (IACUC) of Sichuan PriMed Bio-tech Co., Ltd.

## Authors' contributions

W Zeng, F Gao and L Gong were responsible for designing the experiment and overall investigation. F Gao, X Wen, J Sun, J Liao and W Chen were responsible for coordinate the ultrasound measurement. X Wen was responsible for Doppler ultrasound measurement. J Yang was responsible for anesthesia. X Wen, L Gong and C Qian were responsible for data collection and image analysis. All authors have given final approval of the version to be published. Each author has participated sufficiently in the work to take public responsibility for appropriate portions of the content.

## Author's information

Zeng's group has been studying human disease monkey models for many years. For more information, please visit PriMed website: http://www.scprimed.com.

The Sichuan University group consists of sonographers, radiologists and an anesthetist from the Imaging Center of West China Hospital, Sichuan University. They all have been doing research in cardiovascular disease. See website: http://www.cd120.com/20204/index.jhtml.

## Declarations

Publication charges for this article have been funded by China's Key Projects in the National Science & Technology Pillar Program during the Twelfth Five-year Plan Period (2012BAI23B08).

This article has been published as part of *BioMedical Engineering OnLine *Volume 14 Supplement 1, 2015: Cardiovascular Disease and Vulnerable Plaque Biomechanics. The full contents of the supplement are available online at http://www.biomedical-engineering-online.com/supplements/14/S1
